# Mapping the trigeminal root entry zone and its pontine fibre distribution patterns

**DOI:** 10.1177/0333102420959796

**Published:** 2020-09-22

**Authors:** Alis Guberinic, Veerle Souverein, Ruben Volkers, Anne-Marie van Cappellen van Walsum, Kris CP Vissers, Jeroen Mollink, Dylan JHA Henssen

**Affiliations:** 1 Department of Radiology, Nuclear Medicine and Anatomy, Radboud University Medical Center, Nijmegen, the Netherlands; 2 Donders Institute for Brain, Cognition and Behaviour, Radboud University, Nijmegen, the Netherlands; 3 Department of Anesthesiology, Pain and Palliative Care, Radboud University Medical Center, Nijmegen, the Netherlands; 4 Wellcome Centre for Integrative Neuroimaging, University of Oxford, Oxford, UK

**Keywords:** Anatomy, arrangement, somatotopy trigeminal nerve, trigeminal root, trigeminothalamic tract

## Abstract

**Introduction:**

Recently, an additional trigeminothalamic tract – the dorsal trigeminothalamic tract – has been described in human brainstems by our group next to the known ventral trigeminothalamic tract. As various elements of the trigeminal system are known to be organised in a somatotopic fashion, the question arose whether the fibres within the trigeminal root show specific distributions patterns in their contribution to the ventral trigeminothalamic tract and dorsal trigeminothalamic tract specifically.

**Methods:**

This study investigated the arrangement of the fibres in the trigeminal root by combining various imaging methods in the pons of 11 post-mortem specimens. The pons were investigated by polarised light imaging (PLI) (n = 4; to quantify fibre orientation; 100 µm interslice distance), histochemical staining methods (n = 3; to visualise the internal myeloarchitecture; 60 µm) and ultra-high field, post-mortem magnetic resonance imaging (MRI) (n = 4; for tractography; 500 µm interslice distance).

**Results:**

This study shows that the fibres, from the point where the trigeminal root enters the brainstem, are distinctly arranged by their contribution to the ventral trigeminothalamic tract and dorsal trigeminothalamic tract. This finding is supported by both post-mortem, ultra-high dMRI and different light microscopy techniques.

**Conclusion:**

The data from this study suggest that the fibres in the superior half of the root contribute mainly to the ventral trigeminothalamic tract, whereas the fibres in the inferior half mainly contribute to the dorsal trigeminothalamic tract. Such a somatotopic organisation could possibly create new insights into the anatomical origin of trigeminal neuralgia and the clinical relevance of this somatotopic organisation should therefore be further explored.

## Introduction

The trigeminal nerve (TN) is composed of three main sensory divisions (ophthalmic, maxillary and mandibular) and one motor component. Deficits of the trigeminal system are the cause of numerous acute and chronic neuropathic orofacial pain conditions, including trigeminal neuralgia and trigeminal neuropathic pain conditions (1). Previous research reported on a correlation between the location of vascular compression of the trigeminal nerve and the origin of facial pain (e.g. superomedial compression gave pain in the V1 region) (2). These findings are also supported by the work of Sindou et al. (3), which showed a relationship between the location of conflict of the retro-Gasserian trigeminal root and the distribution of pain. Such functional segregation of the fibres in the trigeminal root has been proposed before (4), although it is regarded as rather controversial (5,6). In general, a gross somatotopic representation of the three major peripheral trigeminal divisions is reasonably well maintained within the trigeminal root, although entwinement of fibres occur as fibres progress from distal to proximal to the brainstem (7). The somatotopy is, however, not exclusive to the trigeminal root and is also maintained in the trigeminal ganglion from its embryological development (8). In the trigeminal ganglion, the afferent fibres of the ophthalmic division are located anteromedially, the mandibular posterolaterally and the maxillary in between (8). The same fundamental spatial organisation is also seen in foetuses of soft-shelled turtles and mouse foetuses (9). Others, on the other hand, specify that somatotopic localisation does not appear to be maintained (10). Nevertheless, improved knowledge on this matter can progress understanding of clinical symptoms, which will aid in diagnostic imaging and more effective treatment (11). Although many studies have contributed to our knowledge about the somatotopy of the peripheral trigeminal nerve, little is known about this segregation within the brainstem.

We therefore investigated the trigeminal root entry zone and pons, to map the arrangement of the trigeminal nerve within the brainstem of four post-mortem samples using polarised light imaging microscopy (PLI). PLI microscopy is capable of quantifying the orientation of fibres and is based on the birefringence of the myelin sheath in histological brain sections (12,13). Findings of the PLI microscopy were thereafter reconstructed by using various advanced neuroimaging techniques, including tractography at 11.7 Tesla (diffusion-weighted) post-mortem magnetic resonance imaging ((d)MRI). This gave a three-dimensional presentation of the trigeminal nerve, further specifying its anatomical and somatotopic basis.

## Methods and materials

### Ethics statement

The brainstems studied herein were part of the anatomical collection of the Department of Anatomy of the Radboud University Medical Center, Nijmegen, the Netherlands. All body donors signed a written informed consent during their lifetime, permitting the use of their body and parts for scientific research and educational purposes. All protocols concerning the acquisition of data and tissue processing were approved by the CMO (Commissie Mensgebonden Onderzoek) region Arnhem-Nijmegen, the Netherlands, and are legislated under Dutch national law (BWBR0005009).

### Specimens

Eleven brains that showed no signs of neurological diseases were included in this study. The brains were extracted from the skull and immersion-fixed using 7.7% formaldehyde at a short post-mortem interval (<48 h). The brains of specimens with a post-mortem interval under 24 h were included for MR scanning, as the post-mortem reduction of the apparent diffusion coefficient (ADC) and fractional anisotropy (FA) is limited in such specimens (14,15). See Table 1 for information on the specimens used in this study.

**Table 1.** Overview of the characteristics of the specimens used in this study.

Specimen	1	2	3	4	5	6	7	8	9	10	11
Imaging modality	Post-mortem MRI	Post-mortem MRI	Post-mortem MRI	Post-mortem MRI	PLI	PLI	PLI	PLI	HW-staining	KB- staining	NS- staining
Age (years)	76	77	62	72	76	63	79	71	87	83	62
Gender	Female	Male	Female	Male	Female	Female	Male	Female	Female	Male	Male
Cause of death	Euthanasia	Pneumonia	Colon cancer	Colon cancer	Liver carcinoma	Euthanasia	Congestive heart failure	Cardiac arrest	Pneumonia	Prostate carcinoma	Lung carcinoma
Time between death and fixation	23 hours	17 hours	12 hours	11 hours	23 hours	18 hours	21 hours	15 hours	×	×	×

HW: Heidenhain-Woelke-staining; KB: Klüver-Barrera staining; MRI: magnetic resonance imaging; NS: Nauta silver staining; × = Not documented; PLI: polarized light imaging.

### Preparation of tissues and magnetic resonance image acquisition

Prior to MR scanning, the four included specimens were soaked in a phosphate-buffered saline solution (PBS 0.1M, pH 7.4) for 7 days in order to reverse the decrease of the T2 relaxation rate of tissue induced by formaldehyde fixation (16). Next, the pontes were placed for 24 hours in Fomblin® (*Solvay Solexis Inc,* https://www.solvay.com/en/brands/fomblin-pfpe-lubricants), a susceptibility-matched, hydrogen-free liquid. Finally, each pons was placed in a 100 ml syringe filled with Fomblin® for MR scanning.

All experiments were performed using an 11.7 T Bruker BioSpec Avance III preclinical MR system (Bruker BioSpin, Ettlingen, Germany) running Paravision 6.0.1, equipped with an actively shielded gradient set of 600 mT/m (slew rate 4570 T/m/sec). A circular polarised resonator was used for signal transmission and an actively decoupled birdcage coil was used for receiving (Bruker BioSpin, Ettlingen, Germany). All scanning was performed at 20 degrees Celsius. Further detailed information concerning the applied MR protocol is provided in Table 2 and has been described extensively before by our group (17).

**Table 2.** Characteristics of the applied 11.7T MRI protocol.

Anatomical imaging with True Fast Imaging with SteadyState Free Precession (TRUFI)	
T_E_	7.0 ms
T_R_	3.3 ms
α	20 degrees
Voxel size	0.25 mm isotropic
Diffusion weighted spin echo planar imaging	
T_E_	30.7 ms
T_R_	13.8 ms
α	30 degrees
Δ	12.5 ms
δ	4.0 ms
Number of directions	256 gradientdirections
Number of averages	2.0
Number of q = 10 mm^−1^ (b = 0 equivalent)	6.0
Voxel size	0.50 mm isotropic
b-value (equivalent)	≈ 4000 s/mm^2^

T_E_: echo time, representing the time from the centre of the radiofrequency pulse to the centre of the echo; T_R_: repetition time, representing the length of the time between the consecutive points on a repeating series of pulses and echoes; α: flip angle, amount of rotation the magnetisation experiences during the application of the radiofrequency pulse; Δ: time between two consecutive pulses; δ: duration of the pulse; b-value: measures the degree of the applied diffusion weighting.

### Probabilistic tractography

Processing of MR data was performed with the software package FSL (18). To correct for eddy current artifacts and displacement between the different diffusion images, an eddy current correction was applied (19). The bedpostX algorithm was used to model multiple fibre orientations (n = 3) at each voxel (20). To delineate the different tracts sprouting from the trigeminal root in our specimens, probabilistic tractography was performed using the Probtrackx2 algorithm (20,21). A manually defined seed mask (i.e. the starting point of tractography) was placed in the trigeminal root in the mean diffusivity (MD) maps by two neuroanatomists separately (DH and AMvCvW). The placed inferior and superior seeding masks were correlated with the PLI and histological sections based on fixed anatomical landmarks. Seed masks were placed on both sides of each trigeminal root. Streamlines were drawn from each seed-voxel (n = 50000 streamlines/voxel). A waypoint mask was placed manually at the level of the Pr5 in order to prevent the inclusion of other large dominating white tracts that course through the pons (22). Final probabilistic tractography results were converted into region of interest (ROI) masks. ROI-masks were converted into colour-coded maps using the vector map, which shows the principal diffusion tensor direction (V1 map).

### Histological tissue processing and polarised light imaging

Histological sectioning of four of the remaining pontes for PLI microscopy was performed in order to optimally visualise all trigeminal fibres sprouting from the trigeminal root coursing in the ventral and dorsal trigeminothalamic tract (VTTH and DTTH, respectively). First, the pontes were bisected midsagitally in order to fit within the maximum field of view of the PLI microscope. Prior to histological sectioning, all pontes were immersed in a 30% sucrose-solution in 0.1M PBS at 4°C for 7 days as cryoprotection to prevent crystallisation during the freezing process prior to sectioning. The specimens were serially sectioned with an HM 450 Sliding Microtome (Thermo Fisher Scientific Inc., Waltham, MA, USA) at a thickness of 100 microns. All sections were mounted on glass and cover-slipped using the mounting medium polyvinylpyrrolidone, creating an interslice distance of 100 µm.

A Zeiss Axio Imager A2 microscope (Carl Zeiss Microscopy LLC, USA) was upgraded with a stationary polariser, a quarter wave plate and a rotating polarise and thereafter used as polarisation microscope. This setup has been described extensively before (17,23) and has been shown to yield a spatial resolution of 4 μm/pixel. PLI post-processing was carried out by in-house written software in MATLAB (MathWorks, Inc., Natick, MA, USA, 1994–2017).

### Histological tissue processing and histochemical staining

Three brainstems were embedded in paraffin prior to sectioning for histochemical myelin staining. The first stain involved Klüver-Barrera staining, which is a commonly used stain to observe myelin under light microscopy (24). The second stain involved Nauta silver stain, which can be used to visualise neurofilaments. This stain can be used to observe neurodegenerative changes of white matter, but can also be used to study myelinated and non-myelinated fibres (25,26). The third stain comprised modified Heidenhain-Woelke stain (data previously published by Mollink et al. (27)), which contributes to optimal visualisation and differentiation between degrees of myelination within white matter fibre bundles (28,29). Sectioning was performed with an LKB 2260 Macrotome (LKB Instruments, Bromma, Sweden). The knife was positioned at a 15 degree angle with respect to the sectioning plane. The tissue was serially sectioned at 4 µm thickness and every 15th slice was kept for staining, resulting in an interplane resolution of 60 µm. Each successful section was mounted and dried overnight in a stove at 37°C. Macrophotographs of the stained sections (referred to hereafter as the histological slices) were taken with a Canon EOS 550D camera using a Canon 100 mm autofocus lens to digitise the data.

Anatomical findings were reported in agreement with the standardised Paxinos-Watson abbreviation system, which has been adopted by the majority of the available anatomical atlases, including the atlases of Paxinos and Huang (30) and Mai et al. (31).

## Results

### Polarised light images

**Figure 1.**
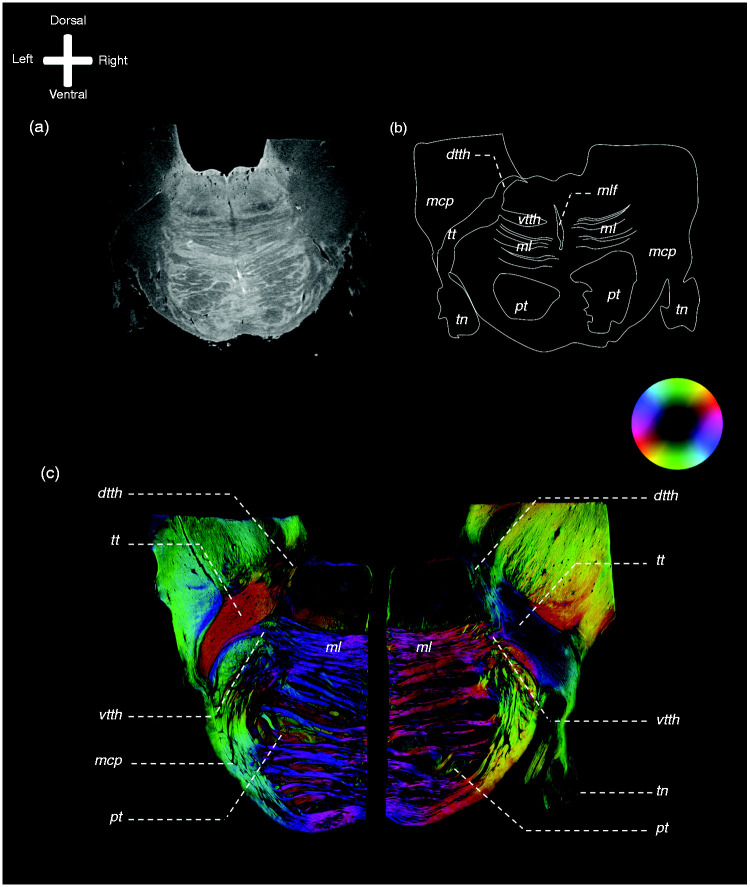
Overview of the anatomy of the pons at the level of the trigeminal entry zone. Exemplary images of MRI and PLI modalities. Anatomical orientation depicted by the anatomical anemone in the above-left corner. The fibre orientation is defined by the colour sphere on the right-hand side of the image. (a) A transverse T2* MR image of the human pons at the level of the entrance of the trigeminal entry zone. (b) Schematic drawing of the same MR image with annotations. (c) PL images of two sides of a bisected brainstem at the level of the trigeminal entry zone. *dtth*: dorsal trigeminothalamic tract; *ml*: medial lemniscus; *pt*: pyramidal tract; *tn*: trigeminal nerve; *tt*: trigeminal tract; *vtth*: ventral trigeminothalamic tract.

**Figure 2.**
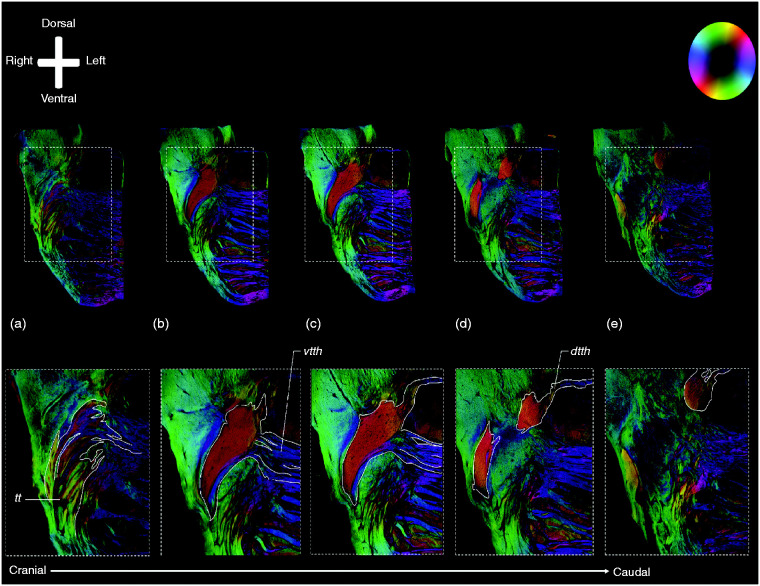
Consecutive polarised light imaging slices showing the fibre distribution patterns of the trigeminal root. Anatomical orientation depicted by the anatomical anemone in the above-left corner. The fibre orientation is defined by the colour sphere in the above-right corner. Figures 2(a)–(e) show consecutive PLI images of the trigeminal nerve. Figures 2(a)–(e) show consecutive PLI images of the trigeminal nerve. (a) is the most rostral part of the trigeminal nerve, (e) represents the most caudal part of the trigeminal nerve. In (a), (b) and (c) one can appreciate the branch coursing to the left, towards the pontine fibres. This branch represents the VTTH. The other branch, which has its trajectory towards the dorsal side of the pons, represents the DTTH. *dtth*: dorsal trigeminothalamic tract; *tt*: trigeminal tract; *vtth*: ventral trigeminothalamic tract.

**Figure 3.**
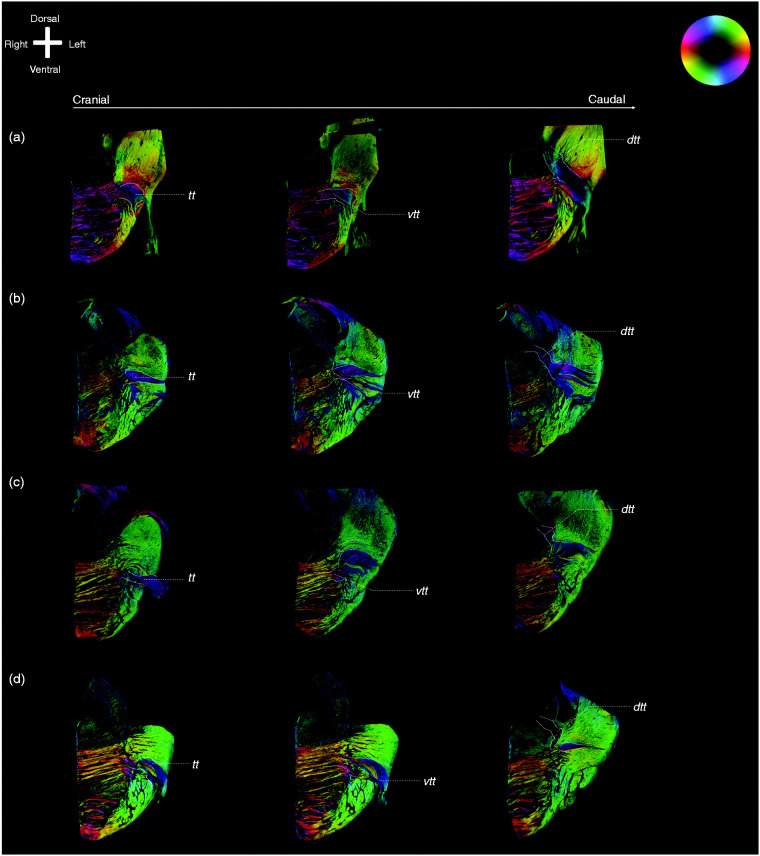
Exemplary images of the trigeminal tracts, the ventral and dorsal trigeminothalamic tracts. Anatomical orientation depicted by the anatomical anemone in the above-left corner. The fibre orientation is defined by the colour sphere on the right-hand side of the image. (a), (b), (c) and (d) represent the four included specimens. For each specimen, three consecutive PLI images of the trigeminal nerve are shown. The four figures on the left side all represent the most rostral part of the trigeminal nerve, while the figures on the right side represent the most caudal part of the trigeminal nerve. The figures in between represent the middle part. The VTTH branch can be seen on the rostral part of the figures, while the DTTH can be seen on the caudal part of the figures. *dtth*: dorsal trigeminothalamic tract; *tt*: trigeminal tract; *vtth*: ventral trigeminothalamic tract.

On the PL images, the trigeminal root can be observed to enter the ventrolateral aspect of each of the collected pontes. Within the pons, the trigeminal tract (TT) can be observed to penetrate the fibres of the middle cerebellar peduncle (MCP) as it courses towards the fourth ventricle. The superior part of the TT fibres can be seen to bend off in the direction of the medial lemniscus (ML). These superior fibres course towards the raphe of the brainstem while partially entwining with the fibers of the ML and are partially bounded ventrally by the ML. These fibres represent the VTTH. The fibres that make up the DTTH sprout from the TT as well and can be seen to course in between the MCP fibres in the direction of the dorsal aspect of the pons. These fibres bend downwards, as they are covered by MCP fibres cranially and continue their course towards the floor of the fourth ventricle. This dorsal division, recognised as the DTTH, runs towards the tegmentum and the fourth ventricle. At the dorsal aspect, after exiting the MCP, the DTTH shows the sprouting of a dispersing tract that runs in the direction of the cerebellum, bounded laterally by the superior cerebellar peduncle. Figure 1 provides a general overview of the anatomy of the pons at the level of trigeminal root entry zone as appreciated on MR and PL images. In Figure 2, consecutive PL images (from A to E) are shown from rostral to caudal. On this figure, it can be appreciated that the trigeminal fibres that course in the upper half of the trigeminal root and TT make up the VTTH. Furthermore, the fibres that contribute to the DTTH are found in the lower half of the trigeminal root and TT in this figure. Figures 2 and 3 depict PL images of consecutive slices of two of the included specimens and the contributions of the trigeminal root to the VTTH and DTTH, respectively.

### Histochemical staining patterns

**Figure 4.**
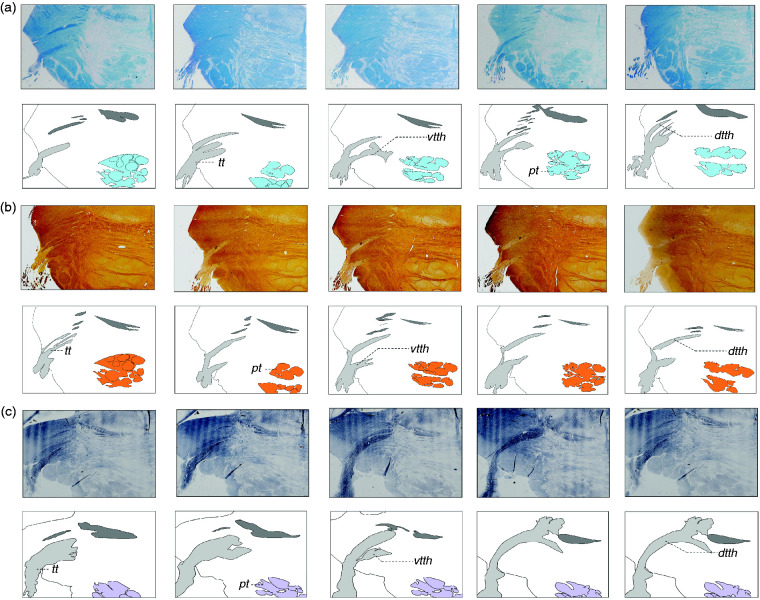
Histochemical staining results. *dtth*: dorsal trigeminothalamic tract; *pt*: pyramidal tract; *tt*: trigeminal tract; *vtth*: ventral trigeminothalamic tract.

The consecutive Heidenhain-Woelke stained sections, the Klüver-Barrera stained sections and the Nauta stained sections (Figure 4) show a similar distribution pattern of the trigeminal root fibres. The upper half of the trigeminal root contributes primarily to the VTTH, whereas the lower half of the fibres course to join the DTTH.

### Probabilistic tractography results

**Figure 5.**
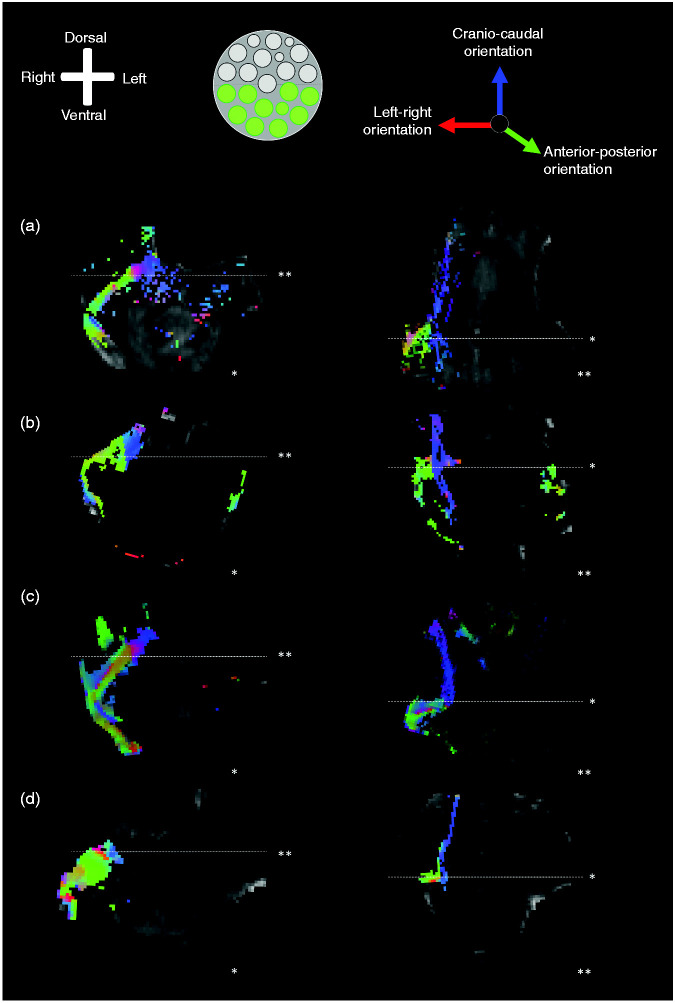
Tractography results when the seeding mask is placed in the superior part of the trigeminal root. Anatomical orientation depicted by the anatomical anemone in the above-left corner. A transection of the trigeminal root showing the definition of the superior part of the trigeminal root. Tractography results of dMRI overlain on 11.7 T mean diffusivity maps. The RGB (red-green-blue) colour cross indicates the principal eigenvector orientations, red = left-right, green = anterior-posterior, blue = cranial-caudal. Tractography results from the superior half TT can be observed to contribute to the VTTH. *Transverse sections (white line shows the level of transection). **Coronal sections (white line shows the level of transection). *tt*: trigeminal tract; *vtth*: ventral trigeminothalamic tract.

**Figure 6.**
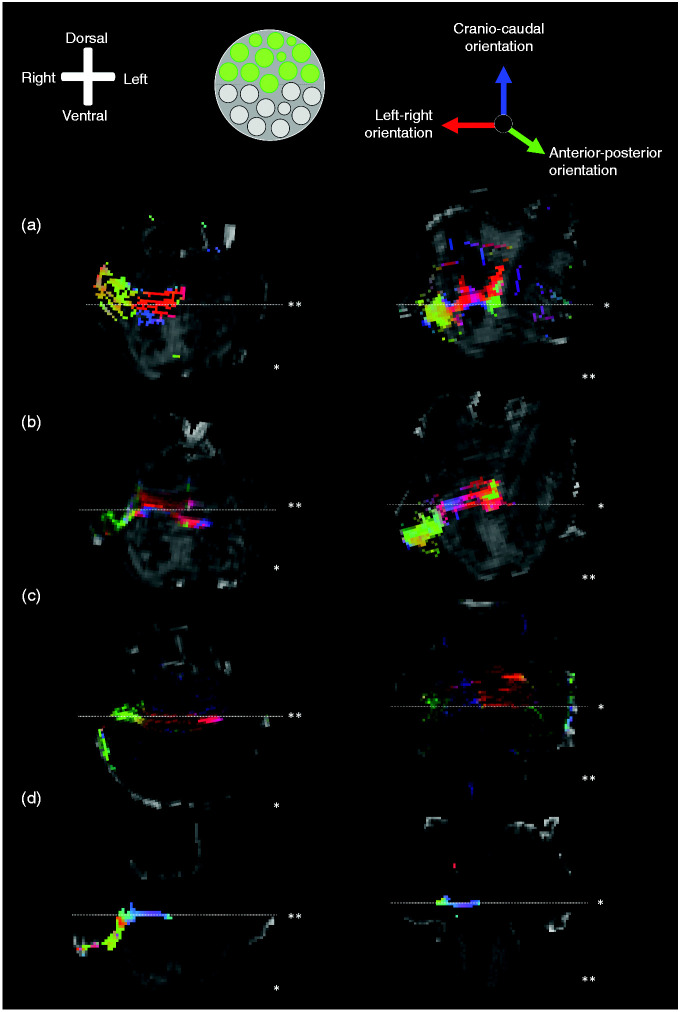
Tractography results when the seeding mask is placed in the inferior part of the trigeminal root. Anatomical orientation depicted by the anatomical anemone in the above-left corner. A transection of the trigeminal root showing the definition of the inferior part of the trigeminal root. Tractography results of dMRI overlain on 11.7 T mean diffusivity maps. The RGB (red-green-blue) colour cross indicates the principal eigenvector orientations, red = left-right, green = anterior-posterior, blue = cranial-caudal. Tractography results from the inferior half TT can be observed to contribute to the DTTH. *Transverse sections (white line shows the level of transection). **Coronal sections (white line shows the level of transection). *dtth*: dorsal trigeminothalamic tract; *tt*: trigeminal tract.

Tractography results confirm the results of the PLI microscopy results. When a seeding mask was placed in the superior half of the trigeminal root, tracts can be seen to course towards the location of the Pr5 within the MCP. From that point, superiorly orientated voxels that lie adjacent to the ML can be appreciated as tracts crossing over to the other side of the brainstem (VTTH) (Figure 5). Tracts seeded from the inferior seeding mask were shown to transverse the MCP. Within the MCP, these tracts bend in a medial direction towards the centre of the brainstem. After emerging from the MCP, the fibres change from a dorsoventral orientation to a craniocaudal orientation. This craniocaudal tract runs in the posterior aspect of the pons near the superior cerebellar peduncle (SCP). These fibres predominantly course on the ipsilateral side of the brainstem (DTTH) (Figure 6).

## Discussion

This study shows that the fibres, from the point where the trigeminal root enters the brainstem, are distinctly arranged by their contribution to the VTTH and DTTH. This finding is supported by both post-mortem, ultra-high dMRI and different light microscopy techniques.

Even though these results show that the superior part of the trigeminal nerve contributes to the VTTH and the inferior part to the DTTH, the controversy regarding whether the somatotopic arrangement of the trigeminal nerve is maintained within the brainstem is not fully answered by these results. The data of the present study do not provide insight into the somatotopic arrangement of the main divisions of the trigeminal nerve (V1, V2 and V3) within the pons. However, Lipari et al. (8) proposed that the spinal nucleus (SN) receives fibres of all three divisions and that the somatotopic arrangement is preserved within the nucleus with the face represented in upside-down fashion. It can therefore be hypothesised that the somatotopic arrangement is preserved in case either the VTTH or the DTTH are responsible for afferent fibres to the spinal nucleus. However, further research will be needed to conclude this. Furthermore, the data show that the bifurcation of the VTTH and DTTH approximates the location of the principal sensory nucleus (PSN). The provided data therefore show the trajectory of the trigeminal tract from the trigeminal root entry zone to the PSN. The course of the two fibre tracts (VTTH and DTTH) originating from the PSN, however, is rather obscure. The classic interpretation is that the trigeminal tract synapses in the PSN and from there projections towards the thalamic nuclei, the trigeminal spinal nucleus and the mesencephalic nucleus arise. However, the classical subdivision that the nociceptive trigeminal input is conveyed by the VTTH and that tactile input is relayed via the DTTH seems to be more complex (32–34). Various authors reported that the fibres that sprout from the dorsal aspect of the PSN (i.e. the DTTH) course towards the ipsilateral posterior ventral nucleus of the thalamus (32–34). This could therefore indicate that the VTTH is responsible for the contralateral and the DTTH for the ipsilateral sensory input to the thalamus. Nevertheless, it remains difficult to assign a functionality to the VTTH and DTTH based on the anatomical results provided here. Another interesting insight is whether these results can improve future therapeutic interventions for trigeminal neuralgia, which will become more target specific. Sindou et al. (2,3), showed that patients suffering from pain in the ophthalmic division are more likely to have a conflict on the superomedial part of the trigeminal root entry zone, which would indicate a somatotopic arrangement of the trigeminal root entry zone. Selective destruction of fibres in the trigeminal root has been proposed by others as a method to relieve pain with only minimal sensory loss (32–35). Although our results do confirm a superior and inferior arrangement, further research is needed to confirm that patients who are undergoing selective destruction of the trigeminal root have fewer consequences than those undergoing non-selective destruction.

### Strengths and limitations

This paper used a multimodality approach to investigate the internal architecture of the trigeminal root and found in all these imaging methods a distribution pattern of the trigeminal fibres that form the origins of the trigeminothalamic connections. Another strength is that, by combining 11.7 T structural and diffusion MRI with PLI microscopy, the imaging resolution from this study bridged from 0.1 mm to 5.0 mm (36). Furthermore, the present study concerns the use of PLI validation (37) and direct implementation of the findings by use of tractography, although this technique is also known to produce false-positive, plausible-looking bundles of white matter anatomy (38). The absence of other imaging methods, including tracer studies, can be regarded as a relative limitation of the present study. Nonetheless, Seehaus et al. showed that tracing studies can be performed in a post-mortem setting on adult human neural tissue over short trajectories (with a maximal length of 13 mm), which could possibly be long enough to observe the distribution of fibres of the trigeminal root in the human brainstem (39). Finally, the precise clinical relevance of this data cannot be determined by the current study protocol and the applied methodologies; for example, due to the fact that the current data provide no insights into the somatotopic arrangement of the trigeminal main divisions in the trigeminal nerve within the pons. Further anatomical research and improved clinical phenotyping of patients suffering from trigeminal neuralgia, combined with new interpretation of radiological data, must be carried out to conclude whether a somatotopic arrangement of the trigeminal nerve is preserved within the pons. Although the sample size was relatively limited, the included specimens showed consistent anatomic features, which decreased the chances of having observed anatomical variants.

## Conclusion

The present study showed that the origins of the VTTH and DTTH can be traced back to specific fibre bundles within the trigeminal root, indicating a distinct organisation of the trigeminal root. The superior half of the trigeminal root was found to contribute mostly to the VTTH, whereas the inferior half of the trigeminal root contains the fibres that constitute the DTTH. Future clinical implications must be further elucidated.

## Article highlights

- The superior half of the trigeminal root was found to contribute mostly to the ventral trigeminothalamic tract (VTTH);
- The inferior half of the trigeminal root contains the fibres that constitute the dorsal trigeminothalamic tract (DTTH).

## Data

All data can be acquired upon reasonable request by contacting the corresponding author.
